# PD57 Patient Involvement In The Development Of Clinical Practice Guidelines For Rare Diseases: A Systematic Literature Review

**DOI:** 10.1017/S0266462324003155

**Published:** 2025-01-07

**Authors:** Anabel Granja Dominguez, Carmen Martín-Gómez, Rocío Rodríguez-López, Lourdes Gonzalez-Bermudez, Juan Antonio Blasco-Amaro

## Abstract

**Introduction:**

One obstacle to the development of clinical practice guidelines (CPGs) for rare diseases (RDs) is the lack of scientific evidence. This can be partially overcome by involving patients in the development of CPGs. Our aim was to develop a process for involving patients with RDs in all stages of CPG development to ensure that their needs and expectations are addressed.

**Methods:**

A literature search was conducted in the MEDLINE, Cochrane Library, and Embase databases and the websites of the European Organization for Rare Diseases, the National Organization for Rare Disorders, and INAHTA. Eligible articles reported methods for involving patients in CPGs, other clinical decision support tools, and research studies. A fit-for-purpose data extraction template was created to capture the following data: author, year, country, type of study, characteristics of the target population, and strategies for participation, engagement, and involvement of patients. Data were synthesized according to methods for recruiting, involving, or engaging patients and obtaining information from them. The entire process was performed by pairs of researchers.

**Results:**

A total of 1,113 records were identified once duplicates were deleted. Of these, 55 were included. The review collected data on types of patients (patient representatives or patient experts) and their recruitment, which could be classified as open or nominated. The various involvement strategies included consultation, participation, and communication. Differences between involving and engaging patients in the CPGs development process were noted. Procedures for obtaining the opinion of patients included surveys, interviews, workshops, and focus groups, among others. The review also provided information on the importance of involving patients in the dissemination and implementation stages of CPG development and the methods for doing so.

**Conclusions:**

When patients with RDs are actively involved in all phases of CPG development, they can contribute to the identification, prioritization, and inclusion of topics pertinent to RDs as questions to be addressed in the CPGs. These aspects might otherwise be overlooked by clinical experts and researchers. Therefore, involving patients with RDs is a promising approach to addressing gaps in the management of these diseases.

